# Supplementary material for: Effects of climate and land-use changes on fish catches across lakes at a global scale
Source: Nat Commun. 2020 May 20;11:2526. doi: 10.1038/s41467-020-14624-2 (PMC7239917; doi:10.1038/s41467-020-14624-2)
Supplement: Supplementary file 1 — Supplementary Information File [file 41467_2020_14624_MOESM1_ESM.pdf]

Supplementary Information

Effects of climate and land-use changes on fish catches across lakes at a global scale

Kao et al.

Supplementary Methods

Data and model inputs associated with fisheries. We obtained data associated with fisheries including annual statistics or estimates for total fish catch, fishing effort, and number of fish stocked, from co-authors, public databases, and literature (Supplementary Data 3). Because data from different sources were usually reported in different units, our model inputs were derived by standardizing data across lakes into the same unit.

Our fish-catch data (Supplementary Figure 1) accounted for the bulk of total reported fish catches across major fishing types in all of the study lakes except Tonle Sap, where comprehensive fish catch and fishing effort estimates were only available for Dai (stationary trawl) fisheries that accounted for about 8% of total reported fish catch[1,2]. Based on lake area given in Supplementary Data 2, we standardized fish catch data into kilogram per hectare, which were then used as our model inputs for the variable CATCH (refer to Supplementary Data 1 for a complete list of symbols used this study). For some lakes, we derived standardized fish catch based on data from a certain part of the lake because of data limitations (Supplementary Data 3). For example, in Lake Titicaca where we were only able to obtain data for the Peruvian waters (60% of the lake area) but not for the Bolivian waters (the rest 40% of the lake area), we derived the standardized fish catch as total Peruvian fish catch divided by the area of Peruvian waters of Lake Titicaca.

Obtained fishing effort data were reported in many different units across and, in some cases, within lakes. As shown in Supplementary Data 3, these units of fishing effort included number of fishing nets, number of commercial fishing licenses, length of gillnet, number of boats, angler days, and so forth. We standardized fishing effort data in each lake into a dimensionless form as

$$\mathrm{EFF}_y = \mathrm{EFFc}_y / \mathrm{EFFcm} \tag{1}$$

where EFF_y is the dimensionless fishing effort in year y and EFFcm is the median of combined fishing effort (EFFc) in the period 1970–2014. The values of EFFy were then used as our model inputs for the variable EFF. For lakes where fishing effort data were reported in multiple units, we followed a previously used method[3], which standardizes each effort type according to a standard effort type associated with the highest fish catch, to derive EFFc as

$$\mathrm{EFFc}_y = \sum\nolimits_t \mathrm{eff}_{y,t} \times \mathrm{CPUE}_t \,/\, \mathrm{CPUEs} \qquad (2)$$

where $\mathrm{eff}_{y,t}$ is the fishing effort of effort type t in year y, CPUE is the average of fish catch per unit effort in the period 1970–2014, CPUEs is the CPUE of the standard effort type, and the ratio of CPUE_t to CPUEs is used as a proxy of fishing power of effort type t relative to standard effort type s. Finally, in lakes where fishing effort data were incomplete, we filled missing values in the Effc time series by linear interpolation, but we did not extrapolate Effc beyond the first or last year when fishing effort data were available in each lake. Time series of fish catch standardized by Effc are given in Supplementary Figure 1.

Obtained data showed that continuous stocking could be important to sustain fish catch in 8 lakes (Lakes Michigan, Simcoe, Chaohu, Kinneret, Taihu, Balaton, Constance, and Geneva), where continuously stocked species contributed >20% of fish catch in >10 years in our study period 1970–2014. For the other lakes, stocking of some species might occur, but those species could either have become self-sustained so that stocking had discontinued or have never been substantial components of fish catch. For example, in Lake Chapala stocked tilapia (*Oreochromis aureus*) had become self-sustained so that the stocking had discontinued. Thus, even though tilapia were an important component of fish catch, we did not include stocking as an explanatory variable for Lake Chapala[4]. In Lake Erie, for example, salmonines have been continuously stocked but the fish catch was dominated by percids and osmerids, so that we did not include stocking as an explanatory variable for Lake Erie either.

For lakes where stocking was important, we standardized total number of fish stocked by lake into a unit of "number of fingerling equivalent per hectare", which were then used as our model inputs for the variable ST. Roughly, fish were stocked across lakes in six different life-history stages, including sac-larvae, larvae (<3 cm), small fingerling (3–5 cm), fingerling (5–10 cm), yearling (10–18 cm), and adult (>18cm). We converted the total number of fish stocked in each life-history stage into an equivalent number of fingerling stocked using conversion factors of (1) 100 sac-larvae = 20 larvae = 2 small fingerling = 1 fingerling[5] and (2) 1 fingerling = 3.5^{-1} yearling = 5^{-1} adult[6]. Finally, we included a time lag to relate the number of fish stocked to when they first contributed to the fish. We assumed it was 2 years for sub-tropic lakes (Lakes

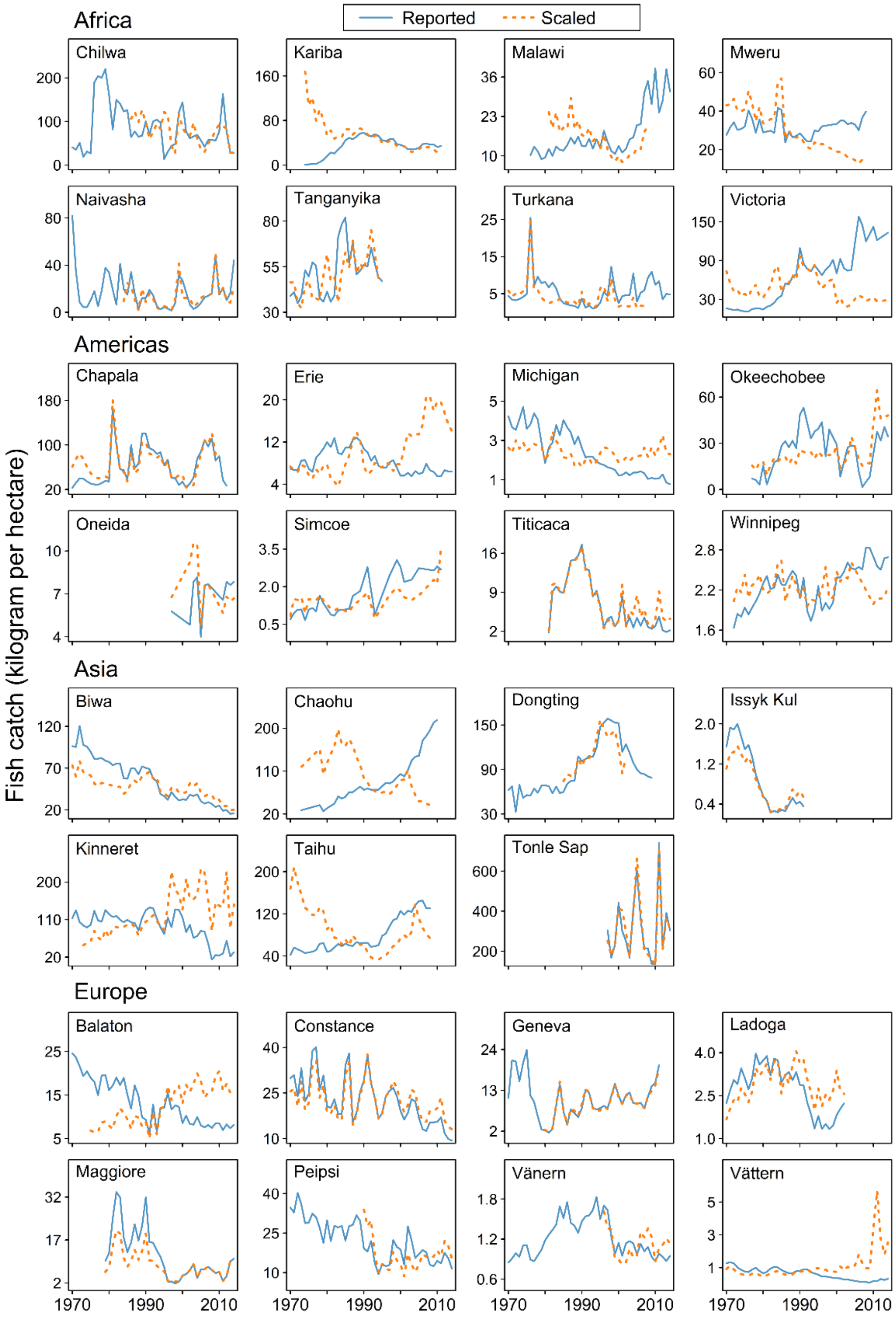

Supplementary Figure 1 Fish catches across the 31 study lakes in the period 1970–2014. Solid lines represent reported fish catches. Dashed lines represent report fish catches scaled by relative fishing effort (with median fishing effort in the period = 1.0). Sources of data used to generate this figure are given in Supplementary Data 3.

Chaohu, Kinneret, and Taihu) and 3 years for north temperate lakes (Lakes Michigan, Simcoe, Balaton, Constance, and Geneva). We assumed that stocked fish contributed to the fish catch sooner in lakes closer to the equator because of higher temperatures and longer growing seasons.

Data and model inputs associated with lake environment. We obtained data associated with lake environment including water temperature, water level, and chlorophyll *a* from co-authors, public databases, and literature (Supplementary Data 3). Our data associated with lake environment could be broadly categorized into three types: (1) long-term monitoring data, which are *in situ* measurements conducted by governmental agencies or research institutions, (2) long-term remote-sensing estimates, which are from public databases developed by governmental agencies or research institutions, and (3) short-term research data, which can be either *in situ* measurements or remote-sensing estimates from the literature. We considered long-term monitoring data and remote-sensing estimates as more reliable than short-term research data because consistent methods were used over time to collect data or derive estimates. As a result, we used short-term research data only for chlorophyll *a* in years when long-term monitoring data and remote-sensing estimates were both unavailable.

Our model inputs for variables WT, WL, and CHL (refer to Supplementary Data 1 for a complete list of symbols used this study) were derived to represent the growing season conditions of fish habitats in each lake, with the limitations of data availability across all lakes. We defined the growing season as May–October for the four high-latitude lakes in Europe (centered at >58°N), April–November for north-temperate lakes (centered between 42°N and 53°N), and the whole year for sub-tropical and tropical lakes (centered between 35°N and 17°S). Our model inputs for the variable WT were the averages of summer surface water temperature. Except for Lake Turkana, we defined summer as July–September for northern hemisphere lakes and January–March for southern hemisphere lakes. We considered Lake Turkana (centered at 3°N) as a southern hemisphere lake because its surface water temperature was always higher in January–March than in July–September[7]. Our model inputs for the variable ΔWL were the year-to-year changes in average water level. Although obtained

water level data were at different temporal resolutions ranging from daily to twice a year (i.e., annual maximum and minimum) across lakes, our annual averages of water level were based on all obtained data within the same year. Our model inputs for the variable CHL were the averages of surface layer chlorophyll *a* during the growing season. Similar to water level data, obtained chlorophyll *a* data were available at different temporal resolutions ranging from several times a month to once a year and our averages of surface layer chlorophyll *a* during the growing season were based on all obtained data within the same growing season.

Data and model inputs associated with climate and land use. We derived inputs for climate and land-use variables in our BNM using data from global, gridded databases for the period 1970–2014. Climate data were available on a monthly basis from the database CRU TS 4.01 (Climatic Research Unit Time Series version 4.01)[8], which has a grid size of 0.5°×0.5° latitude-longitude. Land use data were available on an annual basis from the database LUH2 (Land-Use Harmonization version 2)[9], which has grid size of 0.25°×0.25° latitude-longitude.

Inputs for climate and land-use variables in our BNM were annual catchment-wide averages, which were derived as

$$X_y = \frac{1}{\text{Acat}} \times \sum_g \text{Acat}_g \times X_{y,g} \qquad (3)$$

where X_y is the value of a specific climate or land use variable in year *y*, Acat is the catchment area, Acat_g is the catchment area within grid *g* from a database, and $X_{y,g}$ is the value of *X* in grid *g* in year *y*. To derive Acat and Acat_g, we first delineated catchment of each lake using a Geographical Information System (GIS) ArcGIS 10.4 (www.esri.com/software/arcgis) and a GIS polygon layer HydroBASINS[10], in which global hydrological basins were divided into sub-basin polygons with attributes of flow directions. We delineated the catchment of a lake in GIS as the combination of all the lake's upstream sub-basin polygons subtracted by catchments of all major upstream lakes, as given in Supplementary Data 2. We assumed that major upstream lakes can buffer effects of climate and land use changes in their catchments on the lake of focus. For example, the catchment of Lake Winnipeg was delineated as the whole catchment (over 1 million km^2) subtracted by the catchments (about 471 thousand km^2 total) of two major upstream lakes: Lake Manitoba (surface area 4,720 km^2) and Cedar Lake (surface area 1,320

km^2). $Acat_g$ was derived in GIS by intersecting the GIS polygon layer of 31 catchments with a global polygon layer of 0.5°×0.5° latitude-longitude grids for CRU TS 4.01 or a global polygon layer of 0.25°×0.25° latitude-longitude girds for LUH2.

Model inputs for the variables AT, PRE, and PE (refer to Supplementary Data 1 for a complete list of symbols used this study) were derived using Supplementary Equation (3) and monthly data from the CRU TS 4.01 database. Our model inputs for the variable WT were averages of air temperature in the same summer months as defined above to derive AT. Our model inputs for the variable PRE were the sums of monthly precipitation within a year. Our model inputs for the variable PE were the sums of monthly potential evaporation, which we estimated using Hamon's empirical method[11]:

$$\mathrm{PE}_m = 0.08769 \times \mathrm{ND}_m \times \mathrm{DL}_m \times \mathrm{ES}_m / (\mathrm{Tmx}_m / 2 + \mathrm{Tmn}_m / 2 + 273.15) \quad (4)$$

where PE_m is potential evaporation of month *m* in a unit of m, ND is number of days in a month, DL is average daylight hours in a day, and ES is saturation vapor pressure (millibar), and Tmx and Tmn are maximum and minimum monthly air temperatures (°C) obtained from the CRU TS 4.01 database. DL and ES were empirically estimated[12] as

$$\mathrm{DL}_m = 24 \times \omega_m / \pi \quad (5)$$

$$\begin{aligned}\mathrm{ES}_m = {} & 0.3054 \times \exp[17.27 \times \mathrm{Tmx}_m / (\mathrm{Tmx}_m + 237.3)] + \\ & 0.3054 \times \exp[17.27 \times \mathrm{Tmn}_m / (\mathrm{Tmn}_m + 237.3)]\end{aligned} \quad (6)$$

where ω is sunset hour angle (radian). We estimated ω using an empirical function of latitude and solar declination angle (δ, in radian)[13] as

$$\delta_m = 0.41 \times \sin[2 \times \pi \times (284 + \mathrm{JD}_m) / 365] \quad (7)$$

$$\omega_m = \cos^{-1}[-\tan(\mathrm{LAT}) \times \tan(\delta_m)] \quad (8)$$

where JD_m is the Julian day for the 15th of month *m* and LAT is the latitude.

Model inputs for the variable LUag were derived using Supplementary Equation (3) and annual data from the LUH2 database. There are 13 land-use types in the LUH2 database, including 5 types of cropland, 4 types of undeveloped land, water and ice area, managed pasture, rangeland, and urban land. Our model inputs for the variable LUag were the total percentages of cropland, managed pasture, and rangeland for each catchment.

Data and model inputs associated with socio-economic characteristics. In our correlation analyses, we used two socio-economic characteristics associated with the catchment: access to clean water and shoreline population density. We used the proportion of population using drinking-water and sanitation services in the catchment as a measure for the access to clean water[14]. Data for the proportion of population using drinking-water and sanitation services were available at country level and disaggregated by urban and rural areas[15]. At a catchment scale, we estimated the population using access to (both) drinking-water and sanitation services as

$$\text{POPcat.ac} = \frac{\sum_c \text{POPcat.dr}_c}{\sum_c \text{POPcat}_c} \times \frac{\sum_c \text{POPcat.sa}_c}{\sum_c \text{POPcat}_c} \qquad (9)$$

where POPcat.ac is the proportion of catchment population with access to clean water, subscripted *c* is an index for country, POPcat.dr and POPcat.sa are the catchment population using drinking water and sanitation services, respectively, and POPcat is the (total) catchment population. We estimated POPcat.dr of the country *c* in a catchment as

$$\text{POPcat.dr}_c = \text{POPur.dr}_c \times \text{Acat.ur}_c / \text{Aur}_c + \text{POPru.dr}_c \times \text{Acat.ru}_c / \text{Aru}_c \qquad (10)$$

where POPur.dr and POPru.dr are the population of the country using drinking-water services in urban and rural areas, respectively, and Acat.ur, Acat.ru, Aur, and Aru are urban catchment area, rural catchment area, (total) urban area of the country, and (total) rural area of the country, respectively. Similarly, we estimated POPcat.sa and POPcat of the country *c* in a catchment as

$$\text{POPcat.sa}_c = \text{POPur.sa}_c \times \text{Acat.ur}_c / \text{Aur}_c + \text{POPru.sa}_c \times \text{Acat.ru}_c / \text{Aru}_c \qquad (11)$$

$$\text{POPcat}_c = \text{POPur}_c \times \text{Acat.ur}_c / \text{Aur}_c + \text{POPru}_c \times \text{Acat.ru}_c / \text{Aru}_c \qquad (12)$$

where POPur.sa and POPru.sa are the population of the country using sanitation services in urban and rural areas, respectively, and POPur and POPru are (total) urban and rural population of the country, respectively. We chose to use 2005 data for POPur, POPur.dr, POPur.sa, POPru, POPru.dr, and POPru.sa because it was the first year with relatively comprehensive data[15]. We derived Acat.ur, Acat.ru, Aur, and Aru by using the GIS layer of the 31 catchments developed in this study, a GIS layer of world country (in ArcGIS 10.4), the LUH2 database[9], and ArcGIS 10.4. Note that we considered rural area as the total area of the 12 non-urban land-use types in the

LUH2 database. To be consistent with the estimation of POPcat.ac, we estimated the shoreline population density as the population density within 10 km of a lake's shoreline also in 2005, using population data from a global, gridded database[16], a GIS layer for global lakes[17], and ArcGIS 10.4.

Supplementary References

1. Halls, A. S. & Paxton, B. The stationary trawl (dai) fishery of the Tonle Sap-Great Lake system, Cambodia. in *Inland Fisheries Evolution and Management. Case Studies from Four Continents* (eds Welcomme, R. L., Valbo-Jorgensen, J., & Halls, A. S.) 33–47 (FAO, 2014).
2. Ngor, P. B. et al. Evidence of indiscriminate fishing effects in one of the world's largest inland fisheries. *Sci. Rep.* **8**, 8947 (2018).
3. Lorenzen, K., Almeida, O., Arthur, R., Garaway, C. & Khoa, S. N. Aggregated yield and fishing effort in multispecies fisheries: an empirical analysis. *Can. J. Fish. Aquat. Sci.* **63**, 1334–1343 (2006).
4. Moncayo-Estrada, R., Lyons, J., Escalera-Gallardo, C. & Lind, O. T. Long-term change in the biotic integrity of a shallow tropical lake: A decadal analysis of the Lake Chapala fish community. *Lake Reserv. Manage.* **28**, 92–104 (2012).
5. Gerdeaux, D., Anneville, O. & Hefti, D. Fishery changes during re-oligotrophication in 11 peri-alpine Swiss and French lakes over the past 30 years. *Acta Oecol.* **30**, 161–167 (2006).
6. Kao, Y.-C., Rogers, M. W. & Bunnell, D. B. Evaluating stocking efficacy in an ecosystem undergoing oligotrophication. *Ecosystems* **21**, 600–618 (2018).
7. Odermatt, D., Danne, O., Philipson, P. & Brockmann, C. Diversity II water quality parameters from ENVISAT (2002–2012): a new global information source for lakes. *Earth Syst. Sci. Data* **10**, 1527–1549 (2018).
8. Harris, I., Jones, P. D., Osborn, T. J. & Lister, D. H. Updated high-resolution grids of monthly climatic observations – the CRU TS3.10 Dataset. *Int. J. Climatol.* **34**, 623–642 (2014).
9. Hurtt, G. C. et al. Harmonization of land-use scenarios for the period 1500–2100: 600 years of global gridded annual land-use transitions, wood harvest, and resulting secondary lands. *Clim. Change* **109**, 117–161 (2011).

10. Lehner, B. & Grill, G. Global river hydrography and network routing: baseline data and new approaches to study the world's large river systems. *Hydrol. Process.* **27**, 2171–2186 (2013).

11. Lu, J., Sun, G., McNulty, S. G. & Amatya, D. M. A comparison of six potential evapotranspiration methods for regional use in the southeastern united states. *J. Am. Water Resour. Assoc.* **41**, 621–633 (2005).

12. Harwell, G. R. *Estimation of Evaporation from Open Water—A Review of Selected Studies, Summary of U.S. Army Corps of Engineers Data Collection and Methods, and Evaluation of Two Methods for Estimation of Evaporation from Five Reservoirs in Texas*. (U.S. Geological Survey, 2012).

13. Besharat, F., Dehghan, A. A. & Faghih, A. R. Empirical models for estimating global solar radiation: A review and case study. *Renew. Sust. Energ. Rev.* **21**, 798–821 (2013).

14. United Nations. *Sustainable Development Goal 6 Synthesis Report 2018 on Water and Sanitation*. (United Nations, 2018).

15. United Nations Development Programme (UNDP). *Human Development Report 2016: Human Development for Everyone*. (UNDP, 2016).

16. Klein Goldewijk, K., Beusen, A. & Janssen, P. Long-term dynamic modeling of global population and built-up area in a spatially explicit way: HYDE 3.1. *The Holocene* **20**, 565–573 (2010).

17. Messager, M. L., Lehner, B., Grill, G., Nedeva, I. & Schmitt, O. Estimating the volume and age of water stored in global lakes using a geo-statistical approach. *Nat. Commun.* **7**, 13603 (2016)

Supplementary Notes

Here we acknowledge personal funding supports, data providers, and data sources. This research and Y-C.K. is funded by the U.S. Geological Survey National Climate Adaptation Science Center. Funding support also come from the CERES project (H2020, EU 678193) for I.G.C., COFAA and EDI (Instituto Politécnico Nacional) for R.M.E., Swedish Agency for Marine and Water Management (SWAM) for A.S., Grant LIFE15 NAT/IT/000823 for P.V., GINOP 2.3.2-15-

2016-00004 project: "Establishing the sustainable angling-aimed management of Lake Balaton" for A.W., and National Research Foundation–South African Research Chairs Initiative of the Department of Science and Innovation (Grant No. 110507) for O.L.F.W. We thank Fisheries Research Unit, Department of Fisheries, Malawi for Lake Chilwa and Lake Malawi data; Lake Naivasha Fisheries Department for fish catch data; Central Fisheries Research Institute, Zambia for Lake Kariba and Lake Mweru data; Lake Kariba Fisheries Research Institute, Zimbabwe also for Lake Kariba data; J. Sarvala for Lake Tanganyika catch data; Kenya Marine and Fisheries Research Institute, Turkana Office for Lake Turkana data; National Fisheries Resources Research Institute, Uganda, Tanzania Fisheries Research Institute, and Lake Victoria Fisheries Organisation for Lake Victoria data; Mexico's National Fishery and Aquaculture Commission and the Mexican Institute of Water Technology for Lake Chapala data; New York State Department of Environmental Conservation and Cornell University Brown Endowment for Oneida Lake data; Ontario Ministries of the Environment, Conservation and Parks, and Natural Resources and Forestry, and Lake Simcoe Region Conservation Authority for Lake Simcoe data; IMARPE research team in 1973–1993 (especially to H. Treviño Bernal and R. Alfaro Tapia) for Lake Titicaca data; the Ministry of Agriculture and Rural Development of Israel, Fisheries Department (especially to J. Shapiro and Z. Snovsky) for Lake Kinneret data; Balaton Fish Management Non-Profit Ltd and the General Directorate of Water Management of Hungary for Lake Balaton data; S. Blank and T. Basen for compiling Lake Constance data; SOERE OLA-IS, INRA of Thonon-les-Bains, CIPEL, Direction départementale des Territoires de Haute-Savoie and the Federal Office for the Environment (FOEN), Hydrology Division for Lake Geneva data; Commission for the fishery in the Italian-Swiss waters (CISPP), M. Rogora, M. Ciampittiello, and deeply missed G. Morabito for Lake Maggiore data; Estonian Ministry of Rural Affairs for Lake Peipsi data; and the Societies of Water Conservation for lakes Vättern and Vänern data.
