# Supplementary material for: Effects of climate and land-use changes on fish catches across lakes at a global scale
Source: Nat Commun. 2020 May 20;11:2526. doi: 10.1038/s41467-020-14624-2 (PMC7239917; doi:10.1038/s41467-020-14624-2)
Supplement: Supplementary file 3 — Description of Additional Supplementary Information [file 41467_2020_14624_MOESM3_ESM.pdf]

Description of Additional Supplementary Files

File Name: Supplementary Data 1
Description: List of indices, parameters, and variables

File Name: Supplementary Data 2
Description: Selected information for the 31 study lakes

File Name: Supplementary Data 3
Description: Sources of data associated with lake environment, fish catch, fish stocking, and fishing effort

File Name: Supplementary Data 4
Description: Summary statistics of Bayesian networks model coefficients

File Name: Supplementary Data 5
Description: JAGS code for running Markov chain Monte Carlo (MCMC) simulations.

File Name: Supplementary Data 6
Description: R code associated with the estimation of Bayesian networks model (BNM) coefficients.

File Name: Supplementary Data 7
Description: Source data for Figs. 4a–g, 5a–e, 6a–e, 7, and 8.
