# Supplementary material for: Effects of climate and land-use changes on fish catches across lakes at a global scale
Source: Nat Commun. 2020 May 20;11:2526. doi: 10.1038/s41467-020-14624-2 (PMC7239917; doi:10.1038/s41467-020-14624-2)
Supplement: Supplementary file 11 — Reporting Summary [file 41467_2020_14624_MOESM11_ESM.pdf]

nature research

Corresponding author(s): Yu-Chun Kao

Last updated by author(s): Dec 22, 2019

Reporting Summary

Nature Research wishes to improve the reproducibility of the work that we publish. This form provides structure for consistency and transparency in reporting. For further information on Nature Research policies, see Authors & Referees and the Editorial Policy Checklist.

Statistics

For all statistical analyses, confirm that the following items are present in the figure legend, table legend, main text, or Methods section.

n/a	Confirmed	
☐	☒	The exact sample size (n) for each experimental group/condition, given as a discrete number and unit of measurement
☒	☐	A statement on whether measurements were taken from distinct samples or whether the same sample was measured repeatedly
☐	☒	The statistical test(s) used AND whether they are one- or two-sided<br>*Only common tests should be described solely by name; describe more complex techniques in the Methods section.*
☐	☒	A description of all covariates tested
☒	☐	A description of any assumptions or corrections, such as tests of normality and adjustment for multiple comparisons
☐	☒	A full description of the statistical parameters including central tendency (e.g. means) or other basic estimates (e.g. regression coefficient) AND variation (e.g. standard deviation) or associated estimates of uncertainty (e.g. confidence intervals)
☒	☐	For null hypothesis testing, the test statistic (e.g. F, t, r) with confidence intervals, effect sizes, degrees of freedom and P value noted<br>*Give P values as exact values whenever suitable.*
☐	☒	For Bayesian analysis, information on the choice of priors and Markov chain Monte Carlo settings
☒	☐	For hierarchical and complex designs, identification of the appropriate level for tests and full reporting of outcomes
☒	☐	Estimates of effect sizes (e.g. Cohen's d, Pearson's r), indicating how they were calculated

Our web collection on statistics for biologists contains articles on many of the points above.

Software and code

Policy information about availability of computer code

Data collection	No software were used.
Data analysis	Software used include R version 3.5.2, JAGS version 4.3.0, and ArcGIS version 10.4. As given in our code availability statement, our JAGS code for the main model and R code for coefficient estimation are given in the Supplementary Code files. R code associated with input derivation are available upon request to the corresponding author. We did not have any code associated with ArcGIS.

For manuscripts utilizing custom algorithms or software that are central to the research but not yet described in published literature, software must be made available to editors/reviewers. We strongly encourage code deposition in a community repository (e.g. GitHub). See the Nature Research guidelines for submitting code & software for further information.

Data

Policy information about availability of data

All manuscripts must include a data availability statement. This statement should provide the following information, where applicable:

- Accession codes, unique identifiers, or web links for publicly available datasets
- A list of figures that have associated raw data
- A description of any restrictions on data availability

Data used to derive model inputs for variables associated with lake environment, fish catch, fish stocking, and fishing effort (i.e., WT, ΔWL, CHL, CATCH, ST, and EFF) are either publicly available or available upon requests to corresponding authorities, as given in Supplementary Data 3. Data used to derive model inputs for variables associated with climate and land use (i.e., AT, PRE, PE, and LUag) are from publicly available global databases, as described the Supplementary Methods. Source data used to generate Figs. 4, 5, and 6 are given in Source Data.

Field-specific reporting

Please select the one below that is the best fit for your research. If you are not sure, read the appropriate sections before making your selection.

☐ Life sciences ☐ Behavioural & social sciences ☒ Ecological, evolutionary & environmental sciences

For a reference copy of the document with all sections, see nature.com/documents/nr-reporting-summary-flat.pdf

Ecological, evolutionary & environmental sciences study design

All studies must disclose on these points even when the disclosure is negative.

Study description	The goal of this study is to understand how climate and land-use changes affect lake fish catches at a global scale. We use a Bayesian networks modeling approach to analyze time series data for 31 study lakes over the period 1970–2014 and find that effects of climate and land-uses on fish catches can be either positive or negative across lakes.
Research sample	Data used to derive model inputs for variables associated with lake environment, fish catch, fish stocking, and fishing effort (i.e., WT, ΔWL, CHL, CATCH, ST, and EFF) are either publicly available or available upon requests to corresponding authorities, as given in Supplementary Supplementary Data 3. Data used to derive model inputs for climate and land-use variables are from publicly available global databases: CRU TS 4.01 (Climatic Research Unit Time Series version 4.01) and LUH2 (Land-Use Harmonization version 2).
Sampling strategy	Our sample size is limited by the availability of fish-catch data. Having fish-catch data for 31 lakes, with approximately equal numbers of lakes in Africa (N = 8), the Americas (N = 8), Asia (N = 7), and Europe (N =8), is possibly the best that we can get.
Data collection	We obtained our data from co-authors, public databases, and literature. These data are either publicly available or available upon requests to corresponding authorities, as described in Supplementary Methods and Supplementary Data 3.
Timing and spatial scale	Our data were on an annual basis from 1970 to 2014 for 31 lakes across 5 continents (Africa, the Americas, Asia, and Europe). Annual basis was chosen because fish catch and fishing effort were usually reported on an annual basis. Due to the regional difference in data availability, the 31 study lakes and study period were selected to ensure that we could include approximately equal numbers of study lakes in Africa (N = 8), the Americas (N = 8), Asia (N = 7), and Europe (N =8).
Data exclusions	We did not exclude any data.
Reproducibility	Our results were always reproducible when the same data and code were used.
Randomization	Once again, due to the regional difference in data availability, the 31 study lakes and study period (from 1970 to 2014) were selected to ensure that we could include approximately equal numbers of study lakes in Africa (N = 8), the Americas (N = 8), Asia (N = 7), and Europe (N =8).
Blinding	Our methods for variable derivation and model development were both determined before the true data and results are revealed. The methods for variable derivation were minimally modified because of data limitations. Both of the methods for variable derivation and the structure of Bayesian networks model have not been changed after a discussion among coauthors in March/2018, long before we saw the final results in October/2018.

Did the study involve field work? ☐ Yes ☒ No

Reporting for specific materials, systems and methods

We require information from authors about some types of materials, experimental systems and methods used in many studies. Here, indicate whether each material, system or method listed is relevant to your study. If you are not sure if a list item applies to your research, read the appropriate section before selecting a response.

Materials & experimental systems

n/a	Involved in the study
☒	☐ Antibodies
☒	☐ Eukaryotic cell lines
☒	☐ Palaeontology
☒	☐ Animals and other organisms
☒	☐ Human research participants
☒	☐ Clinical data

Methods

n/a	Involved in the study
☒	☐ ChIP-seq
☒	☐ Flow cytometry
☒	☐ MRI-based neuroimaging
